# Filgrastim prophylaxis in elderly cancer patients in the real-life setting: a French multicenter observational study, the TULIP study

**DOI:** 10.1007/s00520-019-04725-0

**Published:** 2019-03-14

**Authors:** Kamel Laribi, Delphine Badinand, Philippe Janoray, Khaled Benabed, Jean-Loup Mouysset, Elizabeth Fabre, Françoise Monchecourt, Rafik Diab

**Affiliations:** 1 Department of Hematology, Le Mans Hospital, Le Mans, France; 2 Department of Medical Onco-Hematology, Le Mans Hospital, 194 Avenue Rubillard, 72037 Le Mans Cedex 9, France; 3 Department of Radiotherapy Oncology, Hospital La Timone, Marseille, France; 4 Oncology Institute of Bourgogne, Dijon, France; 5 Department of Clinical Hematology, Hospital Côte de Nacre, Caen, France; 6 Public Hospital Center of Cotentin, Cherbourg-en-Cotentin, France; 7 Department of Chemotherapy, Outpatient Unit, Polyclinic Parc Rambot Provençal, Aix-en-Provence, France; 8 Department of Medical Oncology, European Hospital Georges Pompidou, Paris, France; 9 Teva Santé, La Défense, France; 10 Specialized Medical Center of Praz-Coutant, Passy, France

**Keywords:** Chemotherapy-induced neutropenia, Elderly patients, Filgrastim, Primary prophylaxis, Secondary prophylaxis

## Abstract

**Purpose:**

Few studies are currently available among elderly patients, justifying the need for better understanding of daily medical practices in terms of use of growth factors to prevent chemotherapy (CT)-induced neutropenia. The primary objective of this study was to describe the use of filgrastim in the elderly.

**Methods:**

Cancer patients aged 65 years and above, undergoing CT and initiating a prophylactic treatment with filgrastim, were enrolled. Patients were followed according to routine medical practice from filgrastim initiation until the end of the CT or after a maximum of 6 cycles.

**Results:**

One thousand one hundred nineteen evaluable patients were documented in the study (mean age 73.9 ± 6.2 years, 52.1% men). The majority were suffering from solid tumor (73%) with ECOG 0–1 for 80% of them. Approximately two-third had a global risk for FN ≥ 20%, and one third < 20%. Through all CT cycles, no differences were observed between age classes ([65–74], [75–85], or > 85) in dose, duration, and time to first injection from CT start. Most patients (84%) received primary prophylaxis (PP) and 70% were administered during the first CT cycle. The median time from CT start until filgrastim was 4 days. The median duration of filgrastim treatment was 5 days. Dose reductions and CT delays were less frequent in patients receiving PP (4.8% and 7.1% respectively) than secondary prophylaxis (9.2% and 13.3% respectively).

**Conclusions:**

Filgrastim use was consistent with French Market Authorization terms. No difference was shown compared with younger patients. Safety data were consistent with the known safety profile.

**Electronic supplementary material:**

The online version of this article (10.1007/s00520-019-04725-0) contains supplementary material, which is available to authorized users.

## Introduction

Neutropenia is one of the most frequent limiting dose toxicities in cancer patients [1, 2]. It mainly depends on the chemotherapy (CT) regimen and can generate serious life-threatening complications. The incidence of febrile neutropenia (FN) varies from 10 to 57% depending on the chemotherapy protocols [1, 3].

Age is an independent risk factor regarding the development of chemotherapy-induced neutropenia (CIN) in solid and hematologic tumors [4]. According to EORTC’s [3, 5] and ASCO’s [6] guidelines, an age higher than or equal to 65 is an aggravating factor related to FN and must be taken into account when deciding on granulocyte colony-stimulating factors (G-CSF) treatment. In France, median age at the time of diagnosis is 68 years old for men and 65 years old for women and approximately 30% of patients are followed by onco-geriatrics [7]. The general condition associated with age is also a significant risk factor, the physiological age being more relevant than the chronological age to predict the risk of chemotherapy-induced neutropenia [8]. Neutropenic complications are not only more frequent among older patients but also more severe, entailing more numerous and longer hospitalizations as well as a higher mortality rate [9].

In order to prevent these complications, elderly patients are often treated with less aggressive CT protocols or with lower doses whereas (i) age is not a contraindication to the use of standard CT protocols: patients over 65 can profit from the same treatments as younger patients with comparable effectiveness [10]; (ii) controlled clinical trials have shown that the use of low-dose or shorter duration CT decreases the patients’ overall survival [11, 12]; (iii) G-CSF proved to be effective in reducing the incidence of FN, namely with elderly patients [13, 14].

Since elderly patients are excluded from most clinical trials [15], few studies are currently available concerning the management of CIN among this specific population of patients [16, 17]. No study under real conditions of medical practice evaluating the use of filgrastim has been conducted on cancer patients aged 65 or over. The optimum treatment duration with filgrastim and the optimum time for treatment initiation are also not clearly determined.

The aim of this national, observational, and multicenter study is to describe the modalities of use of Tevagrastim® in a real-life setting and also the profile of the patients treated among a cohort of patients aged 65 and over and undergoing anticancer chemotherapy in oncology and onco-hematology.

## Patients and methods

### Study design and patients

From January 2014 to May 2015, cancer patients aged 65 years old or over who were undergoing CT and initiating a prophylactic treatment with Tevagrastim® within a French cancer unit were enrolled. Hospital-based oncologists and onco-hematologists located in metropolitan France were offered to participate in the study. Patients were eligible for inclusion in this multicenter observational study if they had been diagnosed with a solid or hematologic malignancy, were receiving CT with at least 3 cycles scheduled from the time of inclusion in the study, and were starting treatment with Tevagrastim® to prevent CIN (either as primary prophylaxis [PP] or secondary prophylaxis [SP]). Patients were excluded if they had been diagnosed with chronic myeloid leukemia or myelodysplastic syndrome or if they had known contraindications to Tevagrastim®. Oral consent was obtained from each patient included. The study was conducted according to the ethical principles of the Declaration of Helsinki and in accordance with Good Epidemiological Practices. Approvals from the French review boards (Advisory Committee for data processing in Health Research [CCTIRS] and French data protection authority [CNIL]) were obtained.

### Study assessments and endpoints

The primary objective of the study was to describe the conditions of use of filgrastim in the prevention of CIN in elderly patients in a real-life setting. Primary endpoint was administration modalities of filgrastim (e.g., time to treatment start, administration doses and patterns, number of injections per cycle, duration of treatment). Secondary endpoints included incidence of CT delays and CT dose reductions, description of patient’s characteristics, and description of cancer management strategies.

After the patient had given an oral consent, the patient’s data were collected by the participating physicians in a prospective manner in an electronic case report form (eCRF) during an inclusion visit when filgrastim was initiated and during a non-compulsory follow-up visit performed at the end of the chemotherapy or after a maximum of six chemotherapy cycles. This non-interventional study was performed under real conditions of medical practice and standard care and did not entail any additional visit or specific exam for the patient.

### Statistical analysis

The statistical analysis was performed using SAS statistical package version 9.2.

All variables collected in the eCRF and all derived parameters were used in the descriptive statistical analysis. Quantitative variables were analyzed in terms of mean, standard deviation, median, first quartile, third quartile, and extreme values. Binary, categorical, and ordinal parameters were analyzed in terms of number and frequency within the various categories. Conditions of use of filgrastim were described according to the following subgroups: type of prophylaxis (PP vs. SP), age (65–75 years old vs. 75–85 years old vs. > 85 years old), type of tumor (solid vs. hematologic), risk for FN associated with CT protocol (high vs. interim vs. low), and global risk for FN (≥ 20% vs. < 20%). The FN risk categories were those defined by the EORTC guidelines [3].

## Results

### Patient characteristics

**Fig. 1:**
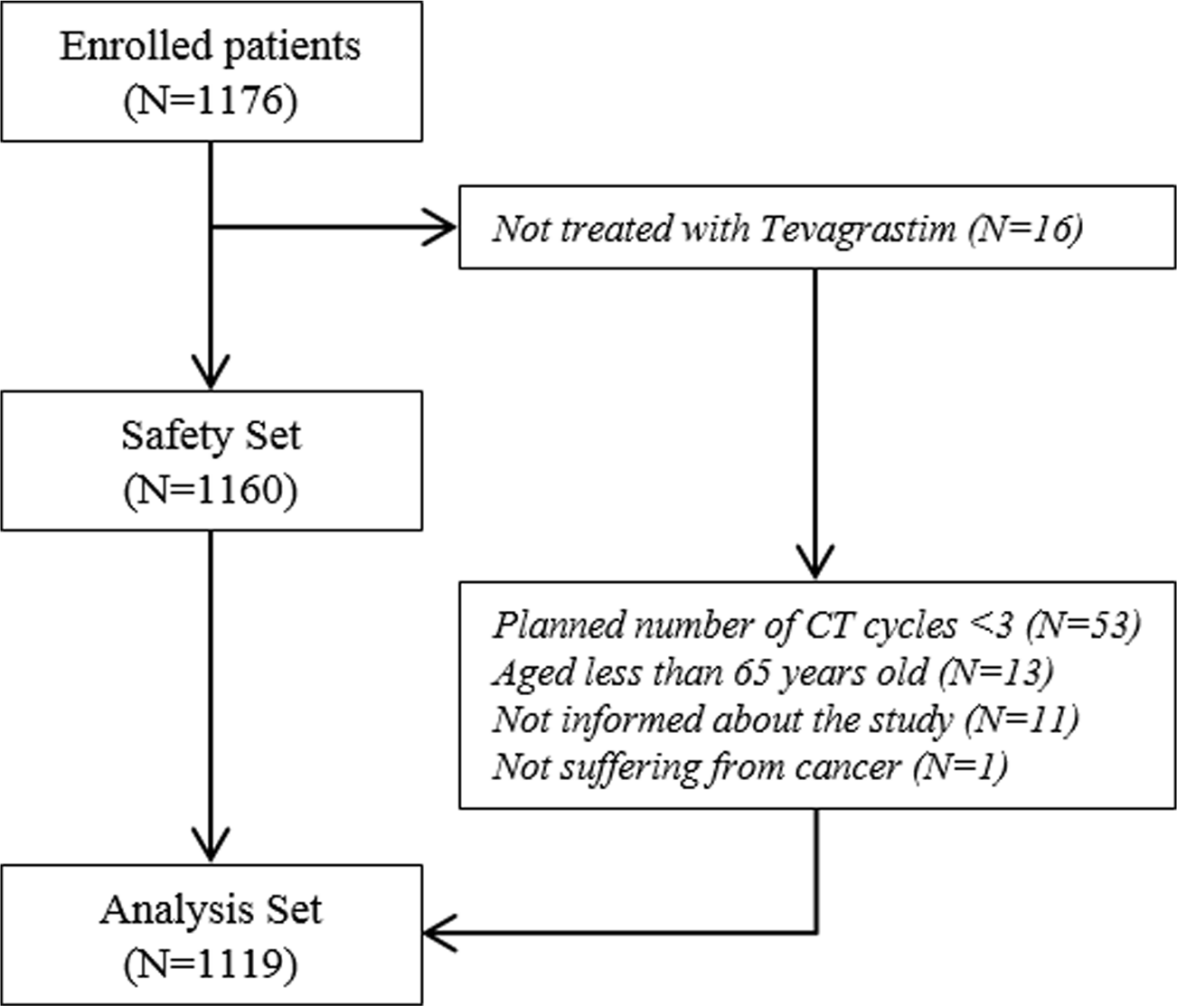
Flow diagram of patient enrollment

A total of 1176 cancer patients were enrolled in the study, 1119 (95.2%) of whom were included in the statistical analysis (Fig. 1). A total of 57 patients were thus excluded from the analysis. The main reason for being excluded from the analysis was an insufficient number of CT cycles to be run (at least 3 cycles were required). Patient main characteristics at inclusion according to the type of prophylaxis (i.e., PP or SP) are summarized in Table 1. Overall mean age was 73.9 ± 6.2 years, 40.9% of patients were at least 75 years old, and 4.4% of patients were at least 85 years old. The majority of patients had a solid tumor (72.9%). Common primary sites for the malignancies included digestive cancer (18.1%), non-Hodgkin lymphoma (18.0%), lung cancer (16.3%), and breast cancer (15.0%). Most patients (79.7%) had a good performance status (i.e., ECOG score 0–1). More than one-third of the patients (35.4%) had received previous chemotherapy, the proportion being slightly higher for secondary prophylaxis patients (43.8% vs. 33.7%). The mean neutrophil count was 3755 ± 1984 cells/mm^3^ at the onset of filgrastim treatment and 89.7% of patients had neutrophil count > 1500 cells/mm^3^. Lower counts were recorded when filgrastim was used as SP rather than PP (2505 ± 1672 cells/mm^3^ vs. 4010 ± 1946 cells/mm^3^). Thirty-four patients (3.2%) presented grade 3 or 4 neutropenia at the onset of filgrastim treatment (11.2% of the secondary prophylaxis patients vs. 1.6% of the primary prophylaxis patients). Overall, each patient presented 1 to 7 risk factors for FN in addition to age (median number of 3): no prophylactic antibiotic treatment (84.5%), advanced stage (51.8%), female gender (47.9%), Hb level < 12 g/dL (41.5%), heart disease (19.7%), malnutrition (13.5%), renal failure (5.7%), history of febrile neutropenia (5.5%). A similar number of risk factors was reported for the patients who received PP or SP although history of severe or febrile neutropenia was more frequent in SP patients (respectively 49.7% and 12.4%).

**Table 1:** Main baseline patient characteristics according to the type of prophylaxis

	Primary prophylaxis (*N* = 934)	Secondary prophylaxis (*N* = 185)	Total (*N* = 1119)
Age (years)			
Mean ± SD	73.9 ± 6.2	73.7 ± 6.1	73.9 ± 6.2
Median (min–max)	73.2 (65–93)	73.3 (65–90)	73.2 (65–93)
≤ 85, *n* (%)	894 (95.7)	176 (95.1)	1070 (95.6)
> 85, *n* (%)	40 (4.3)	9 (4.9)	49 (4.4)
Sex			
Female, *n* (%)	443 (47.4)	93 (50.3)	536 (47.9)
Male, *n* (%)	491 (52.6)	92 (49.7)	583 (52.1)
ECOG score			
0–1, *n* (%)	727 (79.8)	143 (79.0)	870 (79.7)
2, *n* (%)	168 (18.4)	37 (20.4)	205 (18.8)
3, *n* (%)	15 (1.6)	1 (0.6)	16 (1.5)
4, *n* (%)	1 (0.1)	0 (0.0)	1 (0.1)
Tumor site			
Digestive tract, *n* (%)	147 (15.7)	55 (29.7)	202 (18.1)
Non-Hodgkin lymphoma, *n* (%)	182 (19.5)	19 (10.3)	201 (18.0)
Lung or chest, *n* (%)	147 (15.7)	35 (18.9)	182 (16.3)
Breast, *n* (%)	146 (15.6)	22 (11.9)	168 (15.0)
Uterus or ovary, *n* (%)	79 (8.4)	21 (11.3)	100 (8.9)
Prostate, kidney, bladder, *n* (%)	78 (8.3)	13 (7.0)	91 (8.1)
Other site, *n* (%)	155 (16.6)	20 (10.8)	175 (15.6)
Advanced stage^a^	454 (70.2)	126 (85.1)	580 (73.0)
Current chemotherapy			
First line, *n* (%)	662 (71.6)	117 (63.9)	779 (70.3)
Second line, *n* (%)	152 (16.4)	40 (21.9)	192 (17.3)
Third line or higher, *n* (%)	111 (12.0)	26 (14.2)	137 (12.4)
Hb level (g/dL)			
< 12, *n* (%)	370 (40.5)	86 (46.5)	456 (41.5)
≥ 12, *n* (%)	544 (59.5)	99 (53.5)	643 (58.5)
Neutrophil count (cells/mm^3^)			
≥ 1500, *n* (%)	821 (93.6)	126 (70.4)	947 (89.7)
500–1500, *n* (%)	54 (6.2)	52 (29.0)	106 (10.0)
< 500, *n* (%)	2 (0.2)	1 (0.6)	3 (0.3)
Antibiotic prophylaxis	149 (16.0)	24 (13.0)	173 (15.5)
Comorbidities			
Malnutrition, *n* (%)	121 (13.0)	30 (16.2)	151 (13.5)
Immune deficiency, *n* (%)	88 (9.4)	17 (9.2)	105 (9.4)
COPD, *n* (%)	102 (10.9)	25 (13.5)	127 (11.3)
Cardiopathy, *n* (%)	175 (18.7)	45 (24.3)	220 (19.7)
Renal failure, *n* (%)	54 (5.8)	10 (5.4)	64 (5.7)
Hepatic dysfunction, *n* (%)	14 (1.5)	6 (3.2)	20 (1.8%)
Medical history			
History of febrile neutropenia, *n* (%)	39 (4.2)	23 (12.4)	62 (5.5)
History of severe neutropenia^b^	78 (8.4)	92 (49.7)	170 (15.2)
History of fungal infection, *n* (%)	6 (0.6)	4 (2.2)	10 (0.9)
Global risk for FN			
≥20%, *n* (%)^c^	286 (68.1)	53 (58.9)	339 (66.5)
<20%, *n* (%)^c^	134 (31.9)	37 (41.1)	171 (33.5)

^a^For solid tumors only. ^b^Grade 3 or 4. ^c^Among patients with identified risk for FN

### Condition of use of filgrastim

Filgrastim was mainly administered as primary prophylaxis (934 patients, 83.5%) regardless of the ECOG scores. Patients with hematologic malignancies were more likely to receive primary prophylaxis (89.4% vs. 81.3% for solid tumors) while patients presenting with an overall risk of FN < 20% were more likely to receive secondary prophylaxis (21.6% vs. 15.6%). Condition of use of filgrastim according to the type of prophylaxis is detailed in Table 2. In PP patients, filgrastim was initiated within the first CT cycle (79.1%). Approximately 10% of the patients started using filgrastim during or after the third CT cycle.

**Table 2:** Condition of use of filgrastim according to the type of prophylaxis

	Primary prophylaxis (*N* = 934)	Secondary prophylaxis (*N* = 185)	*p* value	Total (*N* = 1119)
Treatment initiation				
First CT cycle, *n* (%)	736 (79.1)	45 (24.5)	< 0.001^a^	781 (70.1)
Second CT cycle, *n* (%)	95 (10.2)	65 (35.3)		160 (14.4)
Third CT cycle or after, *n* (%)	99 (10.6)	74 (40.2)		173 (15.5)
Time to first injection^b^ (days)				
Mean ± SD	4.42 ± 3.53	4.88 ± 5.16	0.118^c^	4.49 ± 3.84
95% CI	4.19; 4.65	4.12; 5.64		4.27; 4.72
Median (min–max)	4 (0–39)	3 (0–31)		4 (0–39)
Daily dose within the first CT cycle (MIU/kg)				
Mean ± SD	0.49 ± 0.09	0.51 ± 0.11	0.322^c^	0.49 ± 0.09
95% CI	0.49; 0.50	0.49; 0.52		0.49; 0.50
Median (min–max)	0.5 (0.3–1.0)	0.5 (0.2–1.0)		0.5 (0.2–1.0)
Route of administration				
Intravenous, *n* (%)	3 (0.3)	0 (0.0)		3 (0.3)
Subcutaneous, *n* (%)	928 (99.7)	184 (100.0)	1.000^d^	1112 (99.7)
Treatment duration per CT cycle (days)				
Mean ± SD	5.42 ± 1.98	4.71 ± 1.49	< 0.001^c^	5.30 ± 1.93
95% CI	5.29; 5.55	4.50; 4.93		5.19; 5.42
Median (min–max)	5 (1–20.3)	5 (2–12.3)		5 (1–20.3)

^a^Chi-square test. ^b^From onset of CT cycle. ^c^Wilcoxon-Mann-Whitney test. ^d^Fisher exact test

The median time before treatment initiation was 4 days after the onset of chemotherapy. In SP patients, filgrastim was mainly initiated after the first CT cycle (75.5%) with more than 40% of the patients who started to receive filgrastim during or after the third CT cycle. In SP patients, the median time before treatment initiation was 3 days after the onset of chemotherapy.

**Fig. 2:**
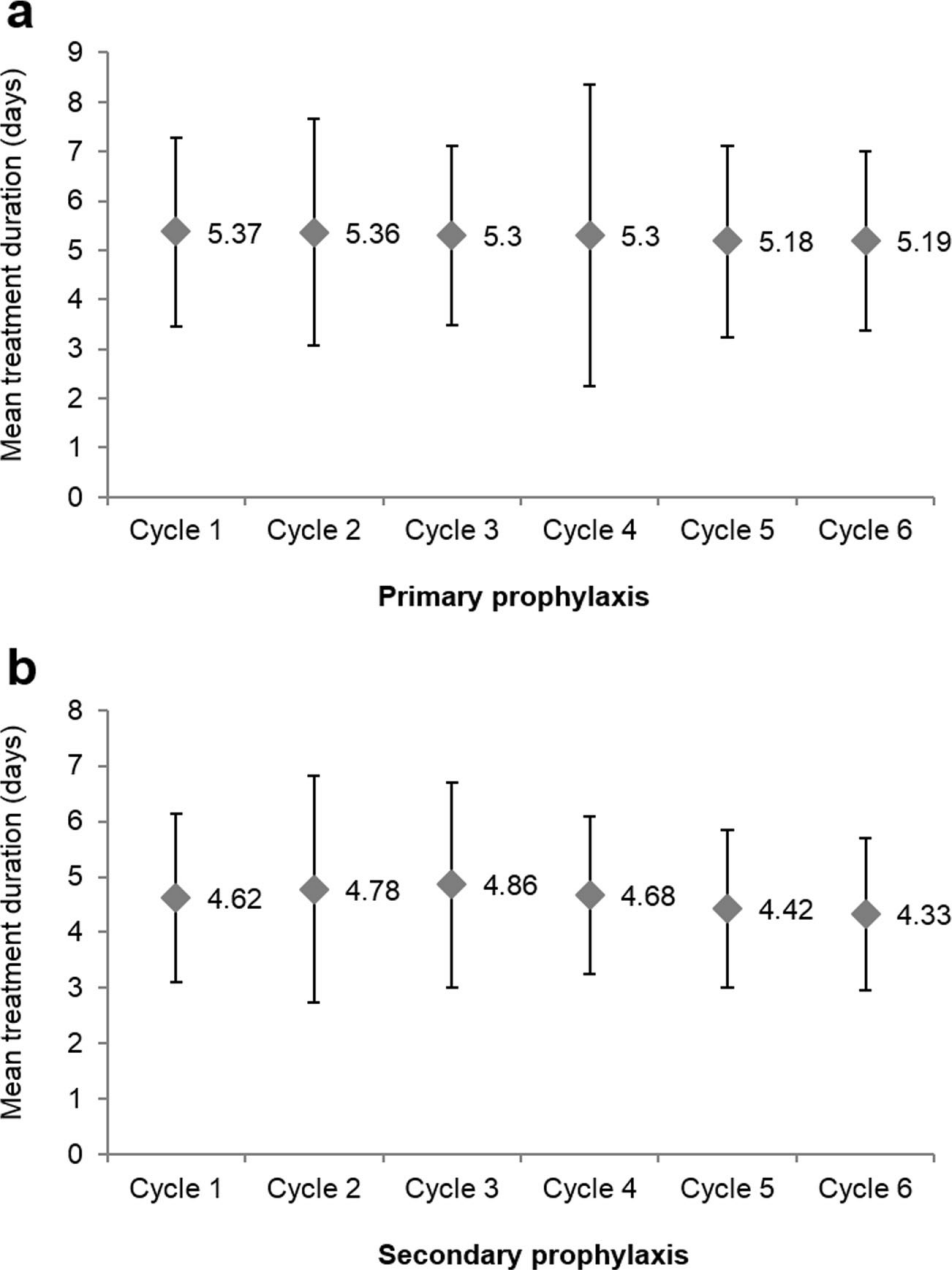
Mean treatment duration (± SD) throughout all CT cycles **a** for primary and **b** secondary prophylaxis. Cycle 1 is the cycle of filgrastim initiation, but not necessarily the first CT cycle

**Fig. 3:**
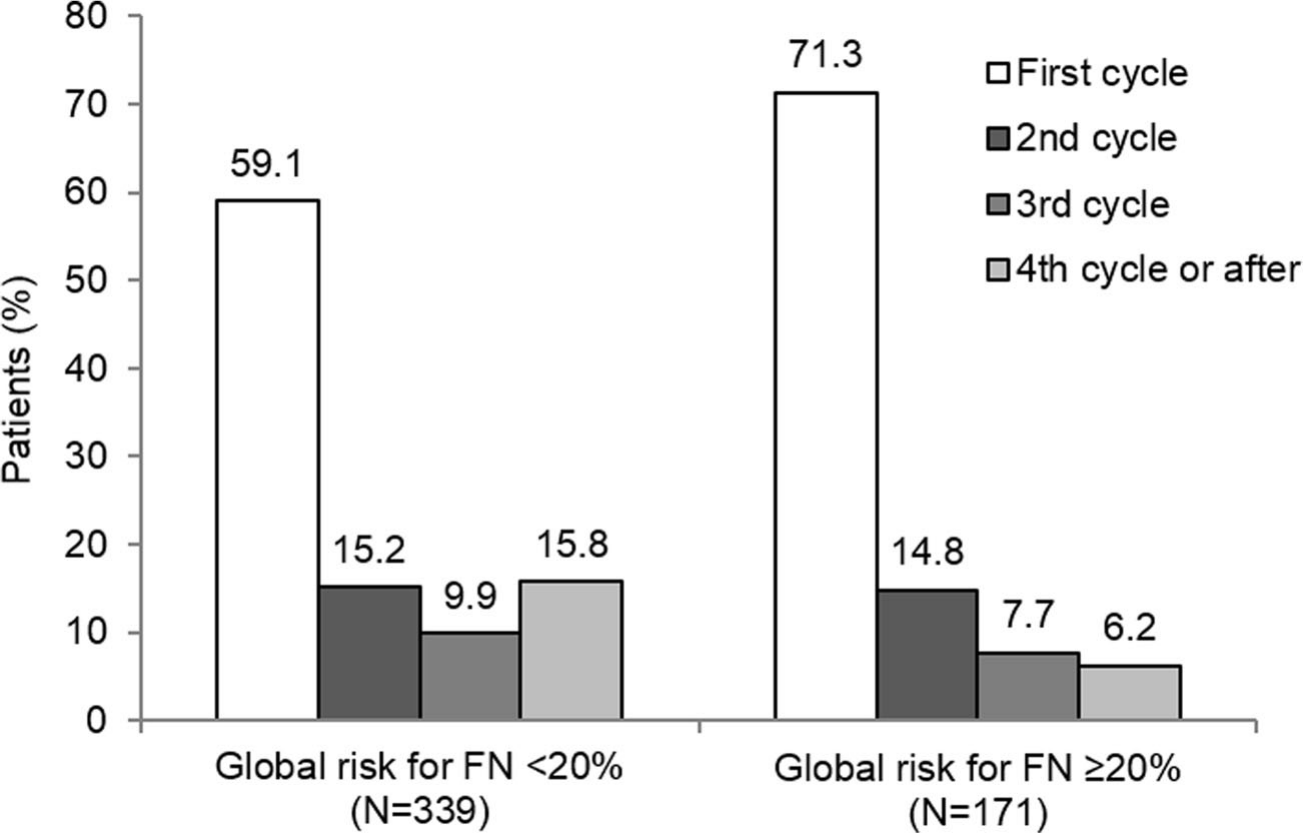
CT cycle involved in filgrastim initiation according to global risk for FN

The preferred dosage, posology, and administration route throughout treatment initiation were identical, regardless of the type of prophylaxis: the median dose was 0.5 MIU/kg/day administered by subcutaneous injections for 99.7% of the patients, and the most commonly used dosage was 30 MIU/0.5 mL. The median treatment duration was 5 days. Throughout all CT cycles, the median time to first injection (from start of CT cycle) was 3 to 4 days for PP patients and 2 to 3 days for SP patients (Online Resource 1) and treatment duration was slightly longer in PP patients compared with SP patients (Table 2 and Fig. 2). Daily dose across CT cycles was not affected by the type of prophylaxis. No main difference was shown regarding treatment modalities according to type of tumor, except the median time to filgrastim injection (from start of CT cycle) that was 6 days for patients with hematologic malignancy and 3 days for patients with solid tumor (Table 3). No difference in condition of use of filgrastim was shown according to age (Online Resource 2). As regards condition of use of filgrastim according to overall FN risk, patients presenting with a high risk level (i.e., ≥ 20%) initiated filgrastim prophylaxis more frequently during the first CT cycle (71.3% vs. 59.1%; *p* = 0.003) (Fig. 3) but mean time to injection, daily dose, and duration of treatment were similar regardless of the risk for FN.

**Table 3:** Condition of use of filgrastim according to tumor type

	Solid tumor (*N* = 816)	Hematologic malignancy (*N* = 303)	*p* value	Total (*N* = 1119)
Type of prophylaxiss				
Primary, *n* (%)	663 (81.3)	271 (89.4)	0.001^a^	934 (83.5)
Secondary, *n* (%)	153 (18.8)	32 (10.6)		185 (16.5)
Treatment initiation				
First CT cycle, *n* (%)	563 (69.4)	218 (71.9)	0.412^a^	781 (70.1)
Second CT cycle, *n* (%)	116 (14.3)	44 (14.5)		160 (14.4)
Third CT cycle or after, *n* (%)	132 (16.3)	41 (13.5)		173 (15.5)
Time to first injection^b^ (days)				
Mean ± SD	4.00 ± 4.15	5.81 ± 2.38	< 0.001^c^	4.49 ± 3.84
95% CI	3.71; 4.29	5.54; 6.08		4.27; 4.72
Median (min–max)	3 (0–39)	6 (0–24)		4 (0–39)
Daily dose within the first CT cycle (MIU/kg)				
Mean ± SD	0.49 ± 0.09	0.49 ± 0.11	0.443^c^	0.49 ± 0.09
95% CI	0.49; 0.50	0.48; 0.51		0.49; 0.50
Median (min–max)	0.5 (0.2–1.0)	0.5 (0.3–0.9)		0.5 (0.2–1.0)
Route of administration				
Intravenous, *n* (%)	3 (0.4)	0 (0.0)		3 (0.3)
Subcutaneous, *n* (%)	809 (99.6)	303 (100.0)	0.567^d^	1112 (99.7)
Treatment duration per CT cycle (days)				
Mean *± S*D	5.21 ± 1.95	5.56 ± 1.84	< 0.001^c^	5.30 ± 1.93
95% CI	5.07; 5.34	5.35; 5.77		5.19; 5.42
Median (min–max)	5.0 (1.0–20.3)	5.0 (3.0–19.0)		5.0 (1.0–20.3)

^a^Chi-square test. ^b^From onset of CT cycle. ^c^Wilcoxon-Mann-Whitney test. ^d^Fisher exact test

### Impact of filgrastim on CT management

CT dose reduction related to neutropenic event was necessary for 58 (5.5%) patients, and on average, each of these patients was subjected to one dose reduction during their study follow-up period. CT delay due to neutropenic event was reported for 85 patients (8.1%). Median duration of CT delay was 7 days. CT dose reductions and CT delays were less frequent in PP patients (4.8% and 7.1%, respectively) than in SP patients (9.2% and 13.3%, respectively). There was no significant difference in the impact of filgrastim treatment on CT management with respect to the age group and the risk level of FN.

### Safety data

A total of 1160 patients received at least one dose of Tevagrastim®. Eighteen patients (1.6%) experienced at least one adverse event (AE) possibly related to Tevagrastim®, and 9 of these patients (0.8%) had at least one serious AE (SAE). Overall, 34 AEs possibly related to Tevagrastim® were reported during the study, primarily general disorders and administration site conditions (7 patients [0.6%]; 9 AEs) and musculoskeletal and connective tissue disorders (6 patients [0.5%]; 10 AEs, mainly bone and back pain). The only AE of particular interest (with respect to the risk management plan for Tevagrastim®) was a lack of efficacy reported in a 65-year-old patient who discontinued treatment due to febrile leukopenia after 8 days of treatment. No immunogenicity assay was performed.

## Discussion

The TULIP study shows that filgrastim is mostly prescribed to elderly patients as primary prophylaxis (PP, 80%). These results are consistent with those of a French study (NEXT study), which reported the use of primary prophylaxis in 91.7% of the patients aged 70 years and older [18]. In contrast, primary prophylaxis was less common in a German study (HEXAFIL) conducted on a younger population (mean age, 59.4 ± 12.7 years), as 59.1% of the patients received secondary prophylaxis [19]. When filgrastim was used as PP, the treatment was predominantly initiated during the first CT cycle (79.1%); only 6.2% of the patients started to receive filgrastim during or after the fourth CT cycle; more particularly, 71.3% of patients with a high risk for CIN (≥ 20%) have initiated filgrastim during the first CT cycle. These results clearly highlight the implementation in common practice of the recommendations of the EORTC (original version written in 2006 and updated in 2010), namely the initiation of G-CSF primary prophylaxis within the first CT cycle for all patients presenting a risk of FN ≥ 20%. A Spanish retrospective study (LEARN study) conducted in 2003 reported a significantly lower proportion of patients treated with primary prophylaxis (45%) [20]. In contrast, a European study (MONITOR-GCSF study), recently conducted on 1447 patients in 12 countries [21], reported a proportion of primary prophylaxis (72.3%) comparable with that of the TULIP study (83.5%). For the patients who received filgrastim as secondary prophylaxis (SP), the treatment was generally initiated after the first CT cycle (75.5% of the cases), likely to address a neutropenic event which occurred during the previous close CT cycles. The median time to treatment initiation was 1 day longer for PP patients compared with SP patients (4 days after the onset of CT cycle vs. 3 days), and 3 days longer for hematologic malignancy compared with solid tumor (6 days vs. 3 days).

In nearly all cases, filgrastim was administered by subcutaneous injections at a daily dose of 0.5 MIU/kg. The treatment was considerably shorter than those usually adopted in randomized trials [22–27]. Mean duration was 5.2 ± 1.9 days for patients with solid tumor and 5.5 ± 1.7 days for patients with hematologic malignancy. According to SmPC of Tevagrastim®, filgrastim treatment must continue after the expected date of the nadir and until the neutrophil counts have returned to normal to induce a lasting response. It is not recommended to discontinue the treatment prematurely before the expected date of the nadir [28]. After a chemotherapy for solid tumors, lymphoma, and lymphocytic leukemia, filgrastim treatment can last up to 14 days [28]. After induction and consolidation treatments for acute myeloid leukemia, the treatment may be significantly longer (up to 38 days) depending on the type, dose, and regimen of the cytotoxic chemotherapy [28]. Nonetheless, clinical studies showed that patients are often treated for shorter periods of time [19, 27, 29, 30]. In a retrospective study conducted from 1998 to 2002 in USA, the mean duration of filgrastim treatment was 6.5 ± 3.1 days for NHL, 6.1 ± 2.9 days for breast cancer, and 4.3 ± 3.1 days for lung cancer [27]. Another American retrospective study conducted from 2004 to 2008 reported treatment durations shorter than 6 days in 74% of patients [31]. In the HEXAFIL study, the median treatment duration was 4 to 5 days depending on the cycles, and the patients treated with PP received longer treatments than those treated with SP (5 days vs. 3 days for the first CT cycle) [19]. On the other hand, identical treatment durations (5 days) were reported in the MONITOR-GCSF study and the TULIP study [21], thus indicating a well-established practice for the prevention of CIN. It should be noted that treatment duration was reported in an overwhelming majority of the patients (less than 0.5% of missing data) in the TULIP study, which contributed to the establishment of reliable conclusions for this parameter.

CT dose reduction and CT delay are main concerns in patients presenting with high-risk neutropenia, especially in elderly patients. When patients develop severe neutropenia (grade 3 or 4) or febrile neutropenia, most of the time, CT dose is reduced or treatments are delayed [32, 33]. These practices have a significant impact on the success rate of the treatments, especially for curative chemotherapies aiming to extend survival or maintain the quality of life. Primary prophylaxis using G-CSF was clearly demonstrated to prevent the development of neutropenia and to reduce the rate of infection and infection-related mortality in adult cancer patients receiving chemotherapy [34, 35]. However, little data is currently available with respect to elderly patients because they are often excluded from randomized trials. Furthermore, randomized trials conducted on Tevagrastim® did not address directly the effects on modifications of the chemotherapy [36–38]. In practice, the rates of CT dose reductions and CT delays due to neutropenic events in patients treated with short-acting G-CSF (filgrastim, lenograstim) vary considerably between studies (ranging from 1 to 46%) [18, 20, 39–43]. In our study, less than 10% of the patients required CT dose reduction or CT delay due to a neutropenic event (5.5% and 8.1%, respectively). These rates were lower for the patients who received PP (4.8% and 7.1%) than for those treated with SP (9.2% and 13.3%). These findings confirm the efficacy of Tevagrastim® for the prevention of CIN in elderly patients in a real-life setting.

There are several limitations to our study. First, the enrolled patients included those with various cancer diagnoses, disease stages, and chemotherapy regimen. Our results therefore cannot necessarily be extrapolated to specific patient populations. Second, we could only determine the risk of CT-induced febrile neutropenia for less than half of the patients included in the analysis, in accordance with the risks defined by the EORTC. Although the risk of developing FN from chemotherapy is a key element in the decision algorithm developed for the prevention of CIN, there is currently no classification system comparing the risk levels of all different protocols of chemotherapy. Whereas the 2010 EORTC guidelines offers a classification for the most common protocols [3], many are not included yet in this classification, therefore leading to a large number of missing data as regards the subgroups analysis for the risk of CIN. Consequently, the description of this parameter remains unclear and the impact of filgrastim treatment modalities on the risk of FN may be difficult to interpret.

Although less robust than clinical trials from the methodological point of view, observational studies have the advantage of enabling “real-life” data collection, and thus to reflect the current clinical practice when a sufficiently large number of patients is included. The TULIP study was conducted on more than 1000 elderly cancer patients (aged 65 years or older). These patients are often excluded from clinical trials and, thus, seldom described in the literature [14]. Therefore, the TULIP study was able to establish the profile of an expanding category of patients as a result of the aging population. In France, more than 700,000 patients each year receive cancer treatments in hospital settings [7]. As of today, the incidence of malignancies after the age of 65 years has increased 11-fold compared with younger adults [44, 45] and nearly 30% of cancer patients are onco-geriatric patients [7]. Hence, it is essential to gain a better understanding of the conditions of use of filgrastim prophylaxis in these patients, which was the goal of the TULIP study.

As a conclusion, the French TULIP study is taking place in a broadly international reflection on cancer management of the elderly patients [44, 45]. Results provide a snapshot of real-life conditions of use of Tevagrastim® in elderly patients who receive prophylactic G-CSF medication: treatment is mainly initiated in primary prophylaxis during the first CT cycle (66%) and mean treatment duration per cycle is 5.3 ± 1.9 days. No difference was observed with regard to age or overall FN risk. Treatment duration was slightly longer when filgrastim was used in primary compared with secondary prophylaxis. The rates of CT delays and CT dose reductions were in the range of those reported previously for younger adult populations. These results highlight the efficacy and safety of prophylaxis with Tevagrastim® as common practice for elderly patients and support the data obtained from previous randomized trials on younger patient populations. Safety data were consistent with the known safety profile.

## Electronic supplementary material

Online Resource 1. Time to first injection throughout each CT cycles according to type of prophylaxis (DOC 47 kb)

Online Resource 2. Condition of use of filgrastim according to age (RTF 166 kb)

Online Resource 3. Chemotherapy protocols of patients with solid tumor (DOC 116 kb)

Online Resource 4. Chemotherapy protocols of patients with hematological malignancy (DOC 42 kb)
