# Supplementary material for: Filgrastim prophylaxis in elderly cancer patients in the real-life setting: a French multicenter observational study, the TULIP study
Source: Support Care Cancer. 2019 Mar 14;27(11):4283–92. doi: 10.1007/s00520-019-04725-0 (PMC6803566; doi:10.1007/s00520-019-04725-0)
Supplement: Supplementary file 1 — Time to first injection throughout each CT cycles according to type of prophylaxis (DOC 47 kb) [file 520_2019_4725_MOESM1_ESM.doc]

 Online Resource 1. Time to first injection throughout each CT cycles according to type of prophylaxis 

 a from onset of CT cycle. b first CT cycle where filgrastim was initiated (could be first CT cycle or not) 
 
