# Supplementary material for: Filgrastim prophylaxis in elderly cancer patients in the real-life setting: a French multicenter observational study, the TULIP study
Source: Support Care Cancer. 2019 Mar 14;27(11):4283–92. doi: 10.1007/s00520-019-04725-0 (PMC6803566; doi:10.1007/s00520-019-04725-0)
Supplement: Supplementary file 2 — Condition of use of filgrastim according to age (RTF 166 kb) [file 520_2019_4725_MOESM2_ESM.rtf]

 Online Resource 2. Condition of use of filgrastim according to age 

a Chi2 test. b from onset of CT cycle. c Kruskal Wallis test. d Fisher exact test 
 
