# Supplementary material for: Filgrastim prophylaxis in elderly cancer patients in the real-life setting: a French multicenter observational study, the TULIP study
Source: Support Care Cancer. 2019 Mar 14;27(11):4283–92. doi: 10.1007/s00520-019-04725-0 (PMC6803566; doi:10.1007/s00520-019-04725-0)
Supplement: Supplementary file 3 — Chemotherapy protocols of patients with solid tumor (DOC 116 kb) [file 520_2019_4725_MOESM3_ESM.doc]

 Online Resource 3. Chemotherapy protocols of patients with solid tumor 

 
 
 
 
 
 

 
