# Supplementary material for: Filgrastim prophylaxis in elderly cancer patients in the real-life setting: a French multicenter observational study, the TULIP study
Source: Support Care Cancer. 2019 Mar 14;27(11):4283–92. doi: 10.1007/s00520-019-04725-0 (PMC6803566; doi:10.1007/s00520-019-04725-0)
Supplement: Supplementary file 4 — Chemotherapy protocols of patients with hematological malignancy (DOC 42 kb) [file 520_2019_4725_MOESM4_ESM.doc]

 Online Resource 4. Chemotherapy protocols of patients with hematological malignancy 

 
